# *Mycoplasma*, *Ureaplasma*, and Adverse Pregnancy Outcomes: A Fresh Look

**DOI:** 10.1155/2010/521921

**Published:** 2010-07-12

**Authors:** Bryan Larsen, Joseph Hwang

**Affiliations:** ^1^Iowa Center for Translational and Clinical Research, Mercy Medical Center, Des Moines University, Des Moines, IA 50312, USA; ^2^Iowa Perinatal Center, Mercy Medical Center, Des Moines, IA 50312, USA

## Abstract

Recent work on the Molicutes that associate with genital tract tissues focuses on four species that may be of interest in potential maternal, fetal, and neonatal infection and in contributing to adverse pregnancy outcomes. *Mycoplasma hominis* and *Ureaplasma urealyticum* have historically been the subject of attention, but *Mycoplasma genitalis* which causes male urethritis in addition to colonizing the female genital tract and the division of *Ureaplasma* into two species, *urealyticum* and *parvum*, has also added new taxonomic clarity. The role of these genital tract inhabitants in infection during pregnancy and their ability to invade and infect placental and fetal tissue is discussed. In particular, the role of some of these organisms in prematurity may be mechanistically related to their ability to induce inflammatory cytokines, thereby triggering pathways leading to preterm labor. A review of this intensifying exploration of the mycoplasmas in relation to pregnancy yields several questions which will be important to examine in future research.

## 1. Introduction

*Mycoplasma hominis, Ureaplasma urealyticum* have had several decades of history among experts in genital tract infectious disease with indications that the former can be part of the normal flora of sexually experienced women and both may play a role in chorioamnionitis, salpingitis, bacterial vaginosis, and postpartum endometritis. Despite an abundance of reports on these organisms, work progressed slowly, mainly due to the fastidiousness and technically challenging culture methods needed to link the organism to clinical conditions. The availability of molecular methods has substantially altered our ability to derive valid information about the pathogenic potential of these bacteria which lack rigid cell walls (Molicutes, specifically the family Mycoplasmataceae).

More recently, interest in *Mycoplasma genitalium* has developed among basic scientists; not so much because of it being an organism that can infect the human reproductive tract—though it can, but because it is a self-replicating microorganism with a minimally sized genome and has been sequenced, showing how little DNA is actually needed to permit microbial life. The genome of this organism is 580,000 base pairs and contains 482 genes. By comparison the genome of *Neisseria gonorrhea* is about 2.2 million base pairs. Despite attention in *Mycoplasma genitalium* being largely to interest in its genetic organization and the focus of the creation of a synthetic Mycoplasma by the J. Craig Venter Institute, it has also been given substantial attention as a genital tract colonizer or pathogen.

In this paper, we will review contemporary information about *Mycoplasma* and *Ureaplasma* with special attention to the manner in which these organisms may be associated with premature birth and related syndromes. As a convenience, at times when both genera are being referred to, the term genital mycoplasmas will be used to denote that the discussion encompasses all the Mycoplasmataceae that may occur in the female genital tract.

## 2. Common Pathways to Preterm Labor and Adverse Pregnancy Outcome

Inflammatory reactions within the genital tract tissues of the pregnant female represent a common pathway that leads to delivery, not only when labor is initiated prematurely, but also when it occurs spontaneously at term. While a complete discussion of the details of parturition could run to a book-length work, just a few points require mention as background for the discussion of the very small bacteria considered here that can contribute to preterm labor and delivery. 

First, the components of parturition include, uterine activity, cervical effacement, and rupture of the fetal membranes, while mechanical processes at one level are ultimately dependent on mediators that are released prior to these mechanical actions occurring. Several sources of proinflammatory substances can be noted including stress on the mother or fetus, blood borne infection of the placenta (even if occult), short cervix allowing vaginal flora to be in abnormally close proximity to the fetal membranes, overdistention of the uterus, and altered vaginal flora in which elevated concentrations of proinflammatory microorganisms may be present. Recent observations indicate that inflammatory cells invade the chorion and amnion in both premature and term labor, and inflammatory cells may be a source of inflammatory mediators [1]. Uterine contractions are driven by prostaglandin in the form of PGF2-a which is increased in the amniotic fluid in preterm labor both when the inflammation is associated with positive microbial culture and even in the absence of positive culture [2].

As a result, there is concern about microbial stimuli that can lead to inflammatory reactions in the gravid uterus as these could initiate the cascade of events leading to precipitous delivery. The source of such inflammation-inducing insults includes specific bacteria whether acquired exogenously (such as STD pathogens) or endogenously (altered ecology of the normal flora), expanding numbers of certain bacteria which are otherwise normal inhabitants of the healthy host, escape of normal bacteria to otherwise privileged sites to anatomic locations in proximity to the fetus, and specific genetic backgrounds of the pregnant woman that allow modified responses to microbial challenge. Any of these might incite inflammation in the cervix, membranes, amniotic fluid, placenta, or cord. Indeed, even the fetus may become part of generating an inflammatory response that may result in preterm labor. 

Thus, attention may be paid to specific organisms, such as the Mycoplasmas and Ureaplasmas, even though they may elicit adverse pregnancy outcomes in ways that are functionally similar to the way other bacteria elicit inflammatory reactions that lead to preterm labor and birth.

Nevertheless, there is a second layer of concern and interest in preterm birth elicited by excursions in microbial populations. If preterm birth occurs secondary to an inflammatory stimulus occurring because of an expanded bacterial population, the infant at the time of birth may be exposed to a qualitatively or quantitatively abnormal bacterial challenge and if premature, this exposure could contribute to the pathologies that are well known among preterm infants. Waites and colleagues noted that the association, though not the actual causality of Ureaplasmas with premature infant bronchopulmonary dysplasia, has well been established [3], and even as more data is collected on etiologic and mechanistic connections, more efficacious and targeted therapies are needed.

## 3. *Mycoplasma genitalium* (MG)

Among sexual partners in a recent study of Mexican American and African American individuals, there was a 9.5 and 10.6% infection rate with MG in women and men, respectively, and symptoms of urethritis among men, but lack of symptoms in women [4]. Further underscoring the role of this organism in male nongonococcal urethritis, an Australian case control study recently published indicated that MG prevalence was 10% among cases versus 2% among controls, but C. trachomatis among cases was 33.5% [5]. 

The organism has also been incriminated as a cause of cervicitis. In a study of a cross section of women attending an STD clinic in Baltimore, the rate of MG in women with cervicitis was 28.6%, while C. trachomatis was 15.8% among cervicitis patients, and MG was found in 19.2% of all patients in the study, while *Chlamydia trachomatis* was present in 11.1%. Although coinfections were common, multiple logistic regression revealed that only MG colonization was significantly associated with cervicitis [6]. Among nonpregnant women, MG also has been associated with salpingitis and in a study of Cefoxitin treatment of salpingitis, failure to eliminate symptoms was attributed to eradicate MG [7]. And in a study of Swedish women undergoing elective termination, postabortal salpingitis was associated with MG colonization in 2.8% of women, furnishing an odds ratio above 6-fold compared to noncolonized women [8]. 

In an animal study, rapid dissemination from vaginal deposition of MG to the upper genital tract and to joints was observed to occur in a mouse model of infection, providing a hint that this mycoplasma may behave through pathogenic pathways similar to other mycoplasmas that have been found in salpingitis [9]. Indeed, a study by Short and coworkers, who studied 22 MG monoinfected women, with pelvic inflammatory disease, comparing them to 172 gonococcal and or chlamydial infected women found natural history and epidemiologic characteristics of the three infections to be similar [10].

The role of MG in premature birth is less well defined and is complicated because it may be superimposed on the carriage of other organisms also implicated as causes or at least associates of adverse pregnancy outcome. A recent case-control study from analyzed pregnant women tested for several genital tract pathogens and after multivariate analysis found that young maternal age and MG colonization were independent risk factors for preterm birth [11]. 

One of the early studies conducted in London looked at more than 1200 pregnant women and found that colonization rate by MG was only 0.7% and only one miscarriage, and no evidence of a connection with preterm birth was discovered. In a study from Japan, investigators also failed to find an association of MG with prematurity, although they did associate *Ureaplasma parvum* (and not *U. urealyticum*) with preterm birth and late spontaneous abortion [12]. In Blanchard and co-workers study, they examined 232 amniotic fluids and were unable to find PCR evidence of MG [13]. Thus, the preponderance of evidence suggests that MG, while quite prevalent, is more important in male urethritis and nonpregnant women than in pregnancy. Nevertheless, indications that MG may have a role in adverse pregnancy outcomes were reported in very recent papers, suggesting that as technology improves and diligence in searching for associations with MG in pregnancy increases, new evidence of its significance may yet emerge.

## 4. *Mycoplasma hominis* and *Ureaplasma urealyticum* (MH and UU)

These two organisms are considered together because much of the literature related to these organisms has developed together. In 1985, a Canadian study by Ebmil and Pereria noted that cervical cultures of MH and UU revealed that the organisms were found simultaneously in women from family planning and prenatal clinics much more frequently than ether was found alone [14]. 

What was first learned about the importance of these organisms in the female genital tract was based on detection by arduous culture methods and in some cases by antibody studies. The early work on these organisms suggested, MH was a marker for sexual activity, with higher prevalence in cervicovaginal cultures of sexually active women than prior to sexual debut. UU was typically thought of as a more virulent organism. These organisms have been associated with bacterial vaginosis and salpingitis, but their role in gynecologic infections has often been a matter of dispute. In a recent survey of women from a sexual health clinic in Australia, the rates of colonization with UU and MH were 6.1 and 13.7%, respectively, however, another *Ureaplasma*, *U. parvum,* was found in 57% of women [15]. 

The term “genital mycoplasmas” is taxonomically imprecise way of referring jointly to MH and UU, and because of its use in the literature, it will also be used in the following paragraphs. Current literature related to the genital mycoplasmas reports on observations that are made both by culture-based detections and by molecular diagnostic methods. Relatively few reports rely on antibodies to these organisms for detection or diagnosis. There is little doubt that molecular methods have revolutionized our understanding of these microorganisms, because when culture and molecular detection are used simultaneously, methods such as PCR seem to offer great sensitivity. As noted by Oh et al. [16], cultivation missed most *Ureaplasma* present in genital tract tissues in women with placental insufficiency. Such individuals would be expected to have a high rate of genital mycoplasma migration into the amniotic fluid or fetal membranes, but by culture, 91% of women with PCR evidence of *Ureaplasma* had negative cultures for the organism [17].

Despite limitations in methods, the preponderance of reports implicates UU more frequently in relationship to prematurity-linked conditions. Thus, for preterm premature rupture of the fetal membranes, preterm labor, intra-amniotic infection, chorioamniointis, funisitis, and placental invasion, the presence of genital mycoplasmas is often interpreted as these organisms having a role in pathogenesis. This may be an overinterpretation, but cautious investigators describe these epidemiologic associations as influencing the risk of adverse pregnancy outcomes. Several reports mentioned below will emphasize the apparent greater importance of UU compared to MH.

It is appropriate to note that in a Czech study of 225 women with pPROM, 68% had cervical colonization by UU compared to 17% among control patients, and 28% of pPROM patients were colonized by MH compared to 15% among control patients [18]. Kasper et al. [19] made an extensive analysis of microbial flora with several categories of vaginal conditions including BV, partial BV, altered vaginal flora, aerobic vaginosis, and genital *Mycoplasma* colonization and reported that after 24 weeks gestation, MH was a risk factor for preterm birth (as were partial BV and abnormal vaginal flora characterized by a diminution of *Lactobacillus*). In contrast, another study [20] of 977 pregnancies in which Nugent scoring was done found that 14% of individuals had a high (8 or greater) Nugent score and this was associated with preterm birth, but genital mycoplasma colonization was not. Interestingly UU colonization was much higher than was high Nugent score (UU was found in 88% and MH was present in only 3%). 

A study of nearly 2000 women in Brussels found a preterm birth rate of 4.9%, and 53.6% of those who delivered prematurely showed UU colonization. In this study, the description of abnormal bacterial flora often accompanied colonization by UU. Although logistic regression showed a significant risk associated with UU, it did not show a commensurate risk associated with what the authors referred to as abnormal flora [21]. Another recent study of 150 women with pPROM reported that UU was present in 96% but was only found in 32% of women who did not experience membrane rupture [22]. Ureaplasma will certainly not be the only threat during pregnancy as noted by a study of bacterial invasion of the amniotic fluid, but it is striking that in 15 women for whom cervical insufficiency was the predisposing cause to amniotic invasion, 7 women had intra-amniotic bacteria (one or more species) and 5 of these 7 had UU [23]. Finally, a recent study of placental cultures from Japan found that among 151 placentas from pregnancies that ended with spontaneous preterm birth before 32 completed weeks, 63 were culture positive for Ureaplasma and 83% of these showed histologic chorioamnionitis, whereas only 30% of Ureaplasma culture negative placentas showed signs of chorioamnionitis [24].

The preceding paragraphs support the idea that both species of genital mycoplasmas may infect the products of conception, but predominantly, UU seems the more frequent and by inference the more virulent of the two opportunistic organisms. This raises further questions as to what constitutes virulence among the genital mycoplasmas and whether virulence can be measured. Further, and probably related, is how the genital mycoplasmas are able to elicit the adverse pregnancy outcomes, and in particular, how may they be related to premature birth?

## 5. *Ureaplasma parvum* (UP)

The taxonomic designation of UP as phylogentically distinct from other mycoplasmas is a relatively recent occurrence and makes backward looks at the literature challenging. It is possible that earlier papers subsumed this organism under UU, but it is also possible that its presence was missed. Therefore, emphasis will be placed in the next paragraphs on the literature that has recognized the separate status of UP.

A modern innovation in microbiology is using the genetic material rather than phenotypic information as a point from which to understand the organism. A ground-breaking paper by Perevre and colleagues [25] compared genomes of MH, MG, and UP and identified 247 coding sequences that were common to the three organisms and for UP there were 280 coding sequences unique to that organism. In addition, analysis of the genomes revealed the energy metabolism, and growth substrates were distinct for the three species. This is notable because it implies that despite living in a common environment, they each derive their energy from the host milieu in different ways.

Given the availability of reagents that can detect UP, it may be expected that a large number of reports will be seen in the future that articulate the ecology of this organism in the human genital tract. For example, a recent report found that in healthy nonpregnant women UP was identified in 57% of their cohort which was far more prevalent than any of the other genital mycoplasmas, a host of viruses or Chlamydia, Trichomonas, or Group B Streptococcus [15]. The organism was confirmed in other populations as well. In a study of women postrenal transplant genital mycoplasmas were more prevalent (40%) than in nontransplant women (27.5%) with UP showing a strong dominance [26]. A group in Poland recently reported on the prevalence of UP in women in relation to cervical pathology and found that among 143 women with squamous intraepithelial lesions compared to 39 healthy controls, mycoplasmas were found in 34% of cases with UP predominating. 

Several reports have focused on the frequency of mycoplasmas including UP in the male genital tract with potential relationships to urethritis, male infertility, and sexual transmission. These topics are not immediately germane, but the literature suggests a role for male partners in female infections.

The arrival of a new taxonomic classification is usually met with the question of what specific role does this newly named organism play in clinical infections? For UP there is emerging evidence that it may play a role in infections of pregnancy or in eliciting conditions associated with prematurity. A recent study indicated that there is a dose-related intra-amniotic inflammatory response to UP and that this is related not only to pPROM, preterm labor, and chorioamnionitis, but also to early onset sepsis in the baby and bronchopulmonary dysplasia [27]. Kataoka's study from 2006 indicated a high prevalence of UP and a statistical association with late abortion and early preterm birth [12].

## 6. Clinical Features of Host-Mycoplasma Interaction and Mechanisms of Adverse Outcomes

The pathogenicity of mycoplasmas in the female genital tract was previously confirmed by the presence of antimycoplasma antibodies among women with intra-amniotic infection and postpartum fevers [28], but currently the details of immunologic networks are better known and it is possible to make more direct links to clinical outcomes. 

Even before specific immunity in the form of antibody is engaged, the host employs mechanisms for recognizing molecular motifs that lead to intracellular signaling and upregulation of host defense factors. A system of recognition factors includes the toll-like receptors or TLRs which have been identified in the genital tract [29]. Activation of TLRs results in the expression of cytokines that can elicit inflammation and phagocytosis leading to antigen presentation and ultimately specific immunity. If we are able to conduct cell culture experiments that indicate that specific molecular motifs known as PAMPs or pathogen-associated molecular patterns exist in genital mycoplasmas, it could explain how these organisms elicit the inflammatory reactions that can lead to labor.

It is appropriate to explore the question of whether genital mycoplasmas have the ability to ligand TLRs with the result of that inflammatory mediators are elaborated. MG is known to upregulate the key signaling molecule NF*κ*B through the Toll 2 and 6 receptors on epithelial cells [16]. This research also indicated vaginal epithelial cells were less responsive than cervical epithelial cells. In a study of detergent-extractable macrophage stimulating activity from UU, activation of Toll 2 and 4 showed activation of a monocyte cell line [30]. Trophoblast cells from term placentas are also activated (producing elevated NF*κ*B and p38 MAP kinase and ERK 1/2 in response to mycoplasma lipoprotein [31]). These trophoblastic cells contain TLRs 2, 4, and 6 and the stimulation through exposure to lipopeptide ultimately elicits production of COX2 and PGE2. Thus, cervical and trophoblast tissue, are able to respond to common elements of mycoplasma, namely the lipopeptide portion of the cell membrane and evidence points to the TLR2 being a key receptor in this process. Cytokines are part of a highly regulated network and include proinflammatory factors as well as anti-inflammatory factors. Proinflammatory cytokines have been associated with amnion and placental infections. Interleukins 1*β*, 6, and 8 as well as TNF-*α* [16, 32], are typically elevated in amniotic fluid, cord blood, and expression in tissues that simultaneously contain bacterial DNA [33]. The cytokines, prostaglandin synthetic pathway (cyclo-oxygenase), and prostaglandins provide a mechanistic connection between the inflammatory stimulus and the ultimate initiation of labor.

## 7. Mycoplasma Virulence

Certainly the substances that elicit an inflammatory response may be considered among the most important virulence factors present in the genital mycoplasmas. However, additional factors may be important in the pathogenic potential of these normally opportunistic organisms. The bulk of the existing literature on this topic relates to the hundreds of mycoplasmas that infect animals, where long standing interest in veterinary medicine has existed. There is evidence to suggest that membrane active substances with hemolytic activity are found in all the arginine using mycoplasmas [34] such as MH. Adherence factors may be predicted as are typical for epithelial microorganisms and like other mucosal pathogens, an IgA protease has been reported for UU [35]. While the difficulty in culturing and working with mycoplasmas in the same way that more conventional organisms are studied has probably limited the pursuit of virulence factors, the annotated genomes of these organisms will allow the prediction of the presence of factors that may be analogous to virulence factors in other organisms and will provide a fertile area of research for theoretical biologists.

## 8. Remaining Issues

The story of mycoplasmas that are found in pregnant women and specifically in the reproductive tract and in occasional association with adverse pregnancy outcome is an incomplete and sometimes confusing story. One complicating factor is the fact that the mycoplasmas can reside in the normal flora and when pregnancy complications that could have a microbial etiology arise, it is often difficult or even illogical to incriminate one microorganism. The difficulty in understanding the mycoplasmas in relation to prematurity is much like the difficulty in connecting preterm birth in women with bacterial vaginosis (BV) to the BV. While the condition has a statistical association with preterm birth, BV itself involves organisms that are part of the normal flora. While the numbers and relationships among the normal vaginal flora organisms are altered in BV, there is a natural tendency among schooled clinicians singling out an individual organism as an object of therapeutic drug treatment, to think that an organism such as *Gardnerella vaginalis* may be the important target when in reality a complex bacterial milieu seems to be important and some bacterial species that may be a part of the process have only been identified relatively recently (*Atopobium vaginae*, for one). 

Realizing that mycoplasmas are also part of the bacterial milieu of BV, we again face the dilemma of whether we can incriminate the mycoplasmas alone for adverse pregnancy outcomes or must consider only the entirety of the flora as responsible. Increasingly, multivariable analysis is being used to tease individual factors out of complex collections of epidemiologic, statistical, and clinical data. In this way, certain organisms that are part of the normal flora can be associated as independent risk factors for clinical conditions. Moreover, it will be important for those who follow this field to pay close attention to the questions being asked and specific associations being hypothesized because there is a difference in whether an association is being made between mycoplasmas and prematurity, and/or low birthweight, and/or amnionitis, and/or amniotic fluid infection, and/or preterm rupture of the membranes. Likewise, the clinical question could be whether one or more of these outcomes is related to genital tract colonization by a particular organism or whether a specific tissue (e.g., amnion, placenta, amniotic fluid) must be infected to result in clinical symptoms. 

Future research will undoubtedly continue to dissect details about the role of mycoplasmas in adverse pregnancy outcome through statistical means and more specific questions. But a limitation of these kinds of investigation will be dependent on the success with which mycoplasmas are identified among cohorts of patients to be studied. Mycoplasmas have been included as a subject of investigation for many years, their presence was first based on culture, and because the culture techniques are challenging, differences in technical expertise between laboratories may have slowed the process of discovery and certainty about the clinical role of these organisms.

Currently, we have the advantage of molecular detection methods. Polymerase chain reaction is now commonly used to detect the presence of these organisms with great specificity, and when used in a quantitative mode has the ability to make inferences about dose response relationships in clinical situations. The ability to exploit molecular methods to examine microbe interactions with their receptors and transduction of cellular signals and upregulation of cytokines and other effector molecules from susceptible tissues puts us on the edge of a clear understanding of the interaction of the genital mycoplasmas and host cells at the most fundamental level. New opportunities for therapeutic interventions should follow understanding infectious mechanisms in extreme detail. 

One last point to be made in the ever increasing emphasis on medicine delivered by health care teams, we should be reminded that the issues of infectious disease leading to preterm birth and other complications of pregnancy do not end with the delivery. The fact is that the infant may be born with infection that threatens survival, but the microorganisms that may help precipitate labor may also participate in other significant problems of the premature infant. The growing literature on this topic is beyond the scope of this paper, but associations of mycoplasmas with bronchopulmonary dysplasia, fetal respiratory distress syndrome [36], and intraventricular hemorrhage are beginning to appear in the literature and should be watched carefully over the coming years. These significant advances in understanding of the consequences of infection should heighten the determination of obstetricians and neonatal intensivists to focus on good communication for the benefit of both mother and her offspring.
